# Forecasting United States heartworm *Dirofilaria immitis* prevalence in dogs

**DOI:** 10.1186/s13071-016-1804-y

**Published:** 2016-10-10

**Authors:** Dwight D. Bowman, Yan Liu, Christopher S. McMahan, Shila K. Nordone, Michael J. Yabsley, Robert B. Lund

**Affiliations:** 1 College of Veterinary Medicine, Cornell University, Ithaca, NY USA; 2 Department of Mathematical Sciences, Clemson University, Clemson, SC USA; 3 Department of Molecular Biomedical Sciences, North Carolina State University, Raleigh, NC USA; 4 Southeastern Cooperative Wildlife Disease Study, Department of Population Health, College of Veterinary Medicine and the Warnell School of Forestry and Natural Resources, The University of Georgia, Athens, GA USA

**Keywords:** Autoregression, CAR Model, Head-banging, Heartworm, Kriging, Prevalence, Spatio-temporal correlation

## Abstract

**Background:**

This paper forecasts next year’s canine heartworm prevalence in the United States from 16 climate, geographic and societal factors. The forecast’s construction and an assessment of its performance are described.

**Methods:**

The forecast is based on a spatial-temporal conditional autoregressive model fitted to over 31 million antigen heartworm tests conducted in the 48 contiguous United States during 2011–2015. The forecast uses county-level data on 16 predictive factors, including temperature, precipitation, median household income, local forest and surface water coverage, and presence/absence of eight mosquito species. Non-static factors are extrapolated into the forthcoming year with various statistical methods. The fitted model and factor extrapolations are used to estimate next year’s regional prevalence.

**Results:**

The correlation between the observed and model-estimated county-by-county heartworm prevalence for the 5-year period 2011–2015 is 0.727, demonstrating reasonable model accuracy. The correlation between 2015 observed and forecasted county-by-county heartworm prevalence is 0.940, demonstrating significant skill and showing that heartworm prevalence can be forecasted reasonably accurately.

**Conclusions:**

The forecast presented herein can a priori alert veterinarians to areas expected to see higher than normal heartworm activity. The proposed methods may prove useful for forecasting other diseases.

## Background

Heartworm disease, caused by the mosquito-borne filarial nematode *Dirofilaria immitis*, is arguably the most medically important parasitic infection of domestic dogs in the United States (US), affecting at least 115,000 dogs in 2015. Beyond the US, heartworm disease is a global veterinary healthcare problem, with *D. immitis* affecting dogs in many parts of South America, Europe, Asia, and Australia [1, 2]. Infection is associated with life-threatening complications and significant financial burden, costing millions in veterinary care annually for disease treatment [3–7]. Although less common and less studied, heartworm disease is also a health concern for other mammals such as domestic cats, domestic ferrets, and some wildlife species [8]. Clinical signs of heartworm disease in domestic dogs include exercise intolerance, coughing, dyspnea, cachexia, anorexia, epistaxis and ascites. Dogs with a high burden of adult heartworms can suffer from pulmonary arterial occlusion and inflammation, leading to pulmonary hypertension and potentially right heart failure. Cats and ferrets may experience similar signs but acute death may occur, even with very low worm burdens. Humans can also be infected with *D. immitis*, but infections are rare, with fewer than 100 cases reported in the US over the last 60 years [9]. Human infection is most commonly asymptomatic, with people considered dead-end hosts for the parasite. While rare, human *D. immitis* infection is highly problematic in that it most often manifests as “coin lesions” in the lungs that may be mistaken for a neoplasm on chest radiographs; surgical excision is necessary to differentiate the two entities [10].

Heartworm disease in dogs is most commonly diagnosed through the detection of circulating *D. immitis* antigen in the blood [3, 11]. The prevalence of heartworm infection in the US varies considerably by geographical region. Two nationwide surveillance studies of *D. immitis* infection seroprevalence (henceforth prevalence) in domestic dogs found the highest prevalence in the Southeast and the lowest in the Northeast [11]. For unknown reasons, a noted decrease in the prevalence of *D. immitis* occurred between the 2001–2007 and 2010–2012 in these studies. Importantly, regardless of time period and even within areas where heartworm infection is considered common, there can be considerable local variation, with prevalence reaching as high as 13 % [12, 13].

Numerous factors are purported to be associated with regional and local variations in *D. immitis* prevalence in domestic dogs. Highly effective commercially available anthelmintics (e.g. macrocyclic lactones (ML) [3], including the avermectins (ivermectin, selamectin) and the milbemycins (moxidectin, milbemycin oxime) can be administered monthly to prevent the development of immature stages into adult worms. Year-round preventive use is recommended throughout the US, yet the majority of dogs only receive seasonal treatment [14]. Even within highly endemic regions, anthelminthic use varies based on client compliance, knowledge, or dog owner’s demographics. In addition, resistance of *D. immitis* to ML has been recently documented and is a growing concern in the Gulf States, but the current extent of resistant phenotypes remains unknown [7, 15]. *Dirofilaria immitis* can be transmitted by over 70 species of mosquitoes, although certain species (e.g. *Aedes trivittatus*, *Aedes sierrensis* and *Culex quinquefasciatus*) are considered more important vectors [16]. Because the density of mosquitoes and community composition of competent vector species is influenced greatly by habitat use and climate, these factors should be considered when investigating factors influencing heartworm disease. In support of this, a previous study found that temperature, median household income, population density, precipitation, elevation, relative humidity, forestation coverage, and surface water coverage all significantly influence *D. immitis* prevalence in dogs [16].

Clearly, it would be advantageous to accurately forecast *D. immitis* prevalence on a local scale, providing an a priori alert to veterinarians in problem areas where immediate remediation measures could be taken. Annual forecasts of emergent infection will also inform veterinary and public health officials to shifting areas of infection, particularly in temperate regions of the US where *D. immitis* is generally absent, rare, or prevalence is highly influenced by annual variation in biotic or abiotic factors.

## Methods

### Data structure

The data studied here contain 31,345,244 heartworm antigen test results from dogs in the conterminous United States from 2011 to 2015, and various climate, geographic and socio-economic factors purported to influence heartworm prevalence. The raw tests were obtained from the Antech and IDEXX laboratories [17, 18]. Over all 5 years in the study, 384,905 of the tests were positive (1.23 %). The test data contain the county/parish of the testing clinic and the month when the tests were conducted; however, no measure of uncertainty is given with the individual test results.

The test data were aggregated into the number of positive and negative tests for each year in each conterminous United States county/parish. Table 1 lists 16 explanatory factors that are purportedly related to dog heartworm prevalence, as well as their time period of record and geographic scale of collection. These 16 factors include the climatic variables of annual temperature, precipitation and relative humidity, the geographic variables of county elevation, forestation coverage and surface water coverage, the socio-economic variables of county population density and median household income, and the presence of eight mosquito vectors. For more details on these factors, see [19].

**Table 1:** Factors purported to influence heartworm prevalence

	Factor	Data period	Scale	Notation	Numerical scale of data
Climate factors	Annual temperature	1895–2015	Climate Division	*X*_*s,*1_(*t*)	Continuous
	Annual precipitation	1895–2015	Climate Division	*X*_*s,*2_(*t*)	
	Annual relative humidity	2006–2015	Climate Division	*X*_*s,*3_(*t*)	
Geographic factors	Elevation	2012	County	*X*_*s,*4_(*t*)	Continuous
	Percentage forest coverage	2012	County	*X*_*s,*5_(*t*)	
	Percentage surface water coverage	2010	County	*X*_*s,*6_(*t*)	
Societal factors	Population density	2011–2014	County	*X*_*s,*7_(*t*)	Continuous
	Median household income	1997–2014	County	*X*_*s,*8_(*t*)	
Mosquito species	*Aedes aegypti*	2008	County	*X*_*s,*9_(*t*)	*X*_*s,k*_ = 1 if present, and *X*_*s,k*_ = 0 otherwise
	*Aedes albopictus*	2012	County	*X*_*s,*10_(*t*)	
	*Aedes canadensis*	2004	County	*X*_*s,*11_(*t*)	
	*Aedes sierrensis*	2004	County	*X*_*s,*12_(*t*)	
	*Aedes trivittatus*	2004	County	*X*_*s,*13_(*t*)	
	*Anopheles punctipennis*	2004	County	*X*_*s,*14_(*t*)	
	*Anopheles quadrimaculatus*	2004	County	*X*_*s,*15_(*t*)	
	*Culex quinquefasciatus*	2004	County	*X*_*s,*16_(*t*)	

For further discussion, including the source of each factor, see [16]

**Fig. 1:**
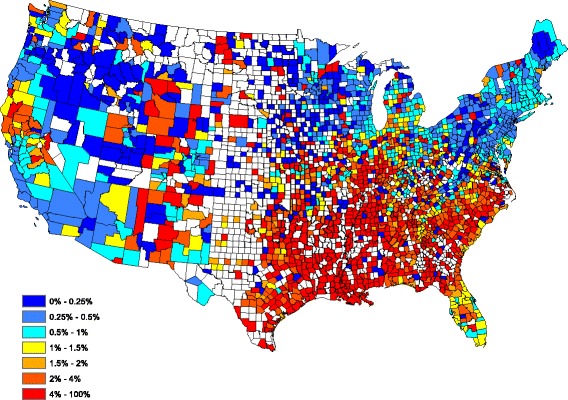
County-by-county raw prevalence aggregated over 2011–2015

Figure 1 displays county-level raw heartworm prevalences obtained by dividing the number of positive tests by the number of tests over all 5 years in the study. The raw prevalences exhibit a large degree of spatial correlation in that neighboring counties tend to report similar prevalences. Significant temporal dependence is also present in the data: the current prevalence is similar to past prevalence. Therefore, this data set requires a statistical model with both spatial and temporal dependence.

**Fig. 2:**
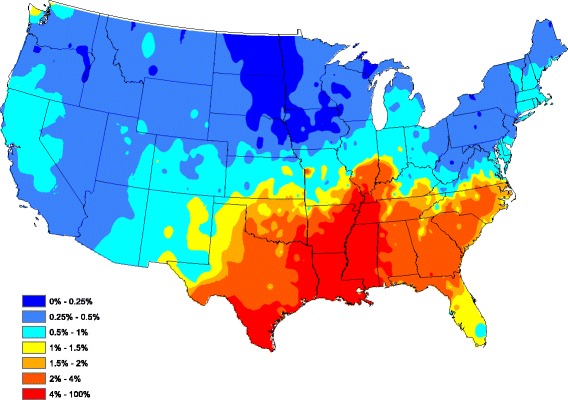
Head-banged baseline map showing heartworm prevalence for an average year during 2011–2015

Figure 2 provides a spatially smoothed prevalence map, using a head-banging smoothing procedure, based on the Fig. 1 prevalences. In the head-banging smoothing procedure, 45 triples were employed. The smoothing was also weighted proportionally to the number of tests in each county over the 5-year period. This prevents the map from signaling a high/low prevalence that is more likely attributed to a small sample (one positive out of three tests has the same prevalence as one hundred positive in 300 tests, though the latter is more indicative of infection risk). Details on head-banging smoothing and its uses in disease mapping are contained in [16]. Figure 2 serves as a contemporary depiction of the “baseline” heartworm risk for dogs in the United States.

### Statistical modeling

The model and methods used to statistically analyze the heartworm tests are now described. The goal here is to assess the significance of the 16 factors and accurately estimate regional heartworm prevalence.

Let *Y*_*s*_(*t*) and *n*_*s*_(*t*) denote the number of positive and total tests conducted in county *s* during year *t*, respectively, for counties *s* ∈ {1, …, *S*} and years *t* ∈ {1, …, *T*}. Methodologies for modeling spatial and temporal dependence have received much recent attention in the statistics literature [20–27]. Among many choices, Bayesian hierarchical models have been prominent due to their flexibility. In a Bayesian hierarchical model, spatial and temporal dependence is modelled in a hierarchy via a series of random effect terms with prescribed structures; see [20, 21] for a modern review of spatio-temporal models. Typically, when modeling the spatial or spatial-temporal dependent count data via parametric models, a Poisson distribution is preferred [21–24]. The following hierarchical regression model is used here:

1 $$ {Y}_s(t)\Big|{n}_s(t),{p}_s(t)\kern1em \sim \kern1em \mathrm{Poisson}\left\{{n}_s(t){p}_s(t)\right\}, $$

2 $$ \log \left\{{p}_s(t)\right\}\kern1em =\kern0.5em {\beta}_0+{\displaystyle \sum_{k=1}^{16}}{\beta}_k{X}_{s,k}(t)+{\xi}_s(t), $$

where log(⋅) denotes natural logarithm, **X**_*s*_(*t*) = (*X*_*s*,1_(*t*), …, *X*_*s*,16_(*t*)) ' is a vector of covariate information for county *s* at time *t* ('denotes matrix transpose), **β** = (*β*_0_, …, *β*_*p*_) ' is a vector of regression coefficients, *p*_*s*_(*t*) denotes the heartworm prevalence of county *s* at time *t*, the symbol ~ means has the distributional type, and ∣ indicates given quantities. Equation (1) indicates that *Y*_*s*_(*t*) has a Poisson distribution with mean *n*_*s*_(*t*)*p*_*s*_(*t*). In addition, it is assumed that the positive test counts (i.e. *Y*_*s*_(*t*)) are conditionally independent of each other given the number of tests, factor information, and random effects. This does not imply that *Y*_*s*_(*t*) is independent across varying space *s* or time *t*.

To relate prevalence to the factors and build spatial and temporal dependence, the model in (2) is proposed, as is common in Poisson regressions [21–24]. There are many ways to induce spatial and temporal dependence from the random effects {*ξ*_*s*_(*t*)}. One natural and popular choice is the conditional autoregressive (CAR) structure

3 $$ \begin{array}{rcl}\kern5em {\xi}_1& =& {\boldsymbol{\upphi}}_1;\\ {}{\xi}_t\Big|{\xi}_{t-1},\varphi & =& \varphi {\xi}_{t-1}+{\boldsymbol{\upphi}}_t,\mathrm{f}\mathrm{o}\mathrm{r}\ t=2,\dots, T;\end{array} $$

4 $$ {\boldsymbol{\upphi}}_t\sim \mathrm{CAR}\left({\tau}^2;\rho \right),\ \mathrm{f}\mathrm{o}\mathrm{r}\ t=1,\dots, T, $$

where **ξ**_*t*_ = (*ξ*_1_(*t*), …, *ξ*_*S*_(*t*)) ' and **ϕ**_*t*_ = (*ϕ*_1_(*t*), …, *ϕ*_*S*_(*t*)) ' are random vectors. Equation (4) indicates that the spatial effects (i.e. **ϕ**_*t*_, for *t* = 1,…,*T*) are independent and identically distributed random vectors that follow a conditional autoregressive (CAR) model [25], which is a popular choice for modeling spatial dependence [26].

More specifically, let **ϕ** = (*ϕ*_1_, …, *ϕ*_*S*_) ' denote a random vector which follows a CAR model. There are several varieties of CAR models. Typically, the CAR model is specified via a series of univariate conditional distributions. Spatial dependence is induced through a neighboring system involving geographically adjacent counties. Our version of the CAR model, taken from [26], uses

5 $$ {\phi}_k\mid {\boldsymbol{\upphi}}_{-k},{\tau}^2,\rho, \mathbf{W}\sim \mathrm{N}\left(\rho \frac{{\displaystyle {\sum}_{i=1}^S}{w}_{k,i}{\phi}_i}{{\displaystyle {\sum}_{i=1}^S}{w}_{k,i}},\kern1em \frac{\tau^2}{{\displaystyle {\sum}_{i=1}^S}{w}_{k,i}}\right),\kern1em \mathrm{f}\mathrm{o}\mathrm{r}\ k=1,\dots, S $$

Here, **ϕ**_− *k*_ = (*ϕ*_1_, …, *ϕ*_*k* − 1_, *ϕ*_*k* + 1_, …, *ϕ*_*S*_) ' is a vector that contains county effects for all counties except the *k*th one and *N*(*μ*, *σ* ^2^) denotes a normally distributed quantity with mean *μ* and variance *σ* ^2^. In addition, **W** = {*w*_*k*,*i*_} is an *S × S* dimensional matrix that describes the neighborhood structure of all counties. Specifically, the entries of **W** are either zero or unity; *w*_*k*,*i*_ = 1 if and only if the *i*th and *k*th counties share some common border.

The parameter *τ* ^2^ in (5) is a scaling variance parameter. In fact, it can be seen that the conditional variance of *ϕ*_*k*_ given its neighbor’s random effects is inversely proportional to the number of counties bordering county *k*. Hence, counties with more neighboring counties tend to have a smaller variance, which is reasonable since data from the bordering counties helps predict the prevalence in the said county.

In Eq. (5), *ρ* ∈ [0, 1] is an autocorrelation parameter that governs correlation between bordering counties. Notice that the conditional expectation of *ϕ*_*k*_ is the weighted arithmetic average of the neighboring random effects, multiplied by *ρ*. When *ρ* = 0, the conditional expectations of *ϕ*_*s*_ are zero and all random effects are independent of each other; antipodally, *ρ* close to unity indicates strong spatial dependence between bordering counties.

In (3), time-dependence is modeled through a temporal autoregressive model of order one (AR(1)), which is a time series staple [28]. Here, it describes prevalence for a fixed county across different years. The parameter φ is the temporal correlation between consecutive years and lies within (−1, 1). This ensures a causal and stationarity solution to the time series model [28], which is needed in estimation.

From (5), it is possible to explicitly identify the joint distribution of **ϕ**, which is multivariate normal:

$$ \boldsymbol{\upphi} \sim \mathrm{N}\left(\mathbf{0},\boldsymbol{\Gamma} \right),\kern0.5em \boldsymbol{\Gamma} ={\tau}^2{\left(\mathbf{D}-\rho \mathbf{W}\right)}^{-1}, $$

where **W** is the previously mentioned neighborhood matrix and **D** = {*d*_*i*,*j*_} is an *S* × *S* diagonal matrix whose *i* th diagonal element is the number of neighboring counties for county *i*.

Bayesian techniques are used to estimate the model parameters, which are **β**, φ, *ρ*, and *τ* ^2^. Thus, to complete the Bayesian hierarchical model, the following prior distributions for these parameters are introduced:

$$ \begin{array}{rrl}\kern1em {\beta}_k& \sim & \mathrm{N}\left(0,1000\right),\kern0.5em \mathrm{f}\mathrm{o}\mathrm{r}\kern0.5em k=0,\dots, 16;\kern1em \\ {}\kern1em \varphi & \sim & \mathrm{Uniform}\left(-1,1\right);\kern1em \\ {}\kern1em \rho & \sim & \mathrm{Uniform}\left(0,1\right);\kern1em \\ {}\kern1em {\tau}^{-2}& \sim & \mathrm{Gamma}\left(0.5,0.05\right).\kern1em \end{array} $$

Prior distributions for φ and *ρ* are taken as uninformative in that all admissible possibilities are equally likely. Priors for the regression coefficients *β*_0_, …, *β*_16_ are taken as diffuse so that inferences for these parameters are based primarily on the data. The prior for *τ* ^− 2^ is chosen as a conjugate prior (the posterior and prior distributions are from the same distributional family) for ease of computation. The random effects and model parameters are estimated based on posterior samples from a Markov chain Monte Carlo (MCMC) simulation. The MCMC simulation for our model uses a combination of Gibbs and Metropolis-Hastings steps. In the implementation of the algorithm, the test data for non-reporting counties was viewed as being latent and was subsequently sampled along with the model parameters. To run our MCMC simulation and assess significance of model parameters, a program was developed and implemented in **R** and **C**++.

## Results

### Model assessment

The spatio-temporal Poisson regression model in (1) has 16 explanatory factors, all of which may not have predictive power. To assess this issue, a full model with all 16 factors was first fitted. Credible intervals, Bayesian analogs to confidence intervals in frequentist statistics, were then created for the parameters of interest. Table 1 summarizes our full model findings, showing 16 regression coefficients estimates (posterior median) and their 95 % highest posterior density (HPD) intervals; for further details about credible and HPD intervals, see [29, 30].

Table 2 implies that not all factors are significant, e.g. 95 % HPD intervals of annual precipitation, elevation, percentage surface water coverage, and all mosquito presence factors except *A. albopictus* contain zeroes. To develop a parsimonious model with only significant factors, all explanatory variables whose 95 % HPD intervals contain zeroes were removed and the model was refitted. This leaves a “reduced model” with the six explanatory factors: annual temperature, annual relative humidity, percentage forest coverage, population density, median household income and *A. albopictus* absence/presence. Parameter estimates (posterior median) and 95 % HPD intervals for the regression parameters for the reduced model are shown in Table 3. The estimates (posterior median) of the other model parameters are φ = 0.914, *ρ* = 0.998, and *τ* ^2^ = 0.802.

**Table 2:** Parameter estimates from the full model

Factor	Estimate	95 % HPD interval
Annual temperature	0.052	[0.038, 0.066]
Annual precipitation	0.008	[-0.031, 0.047]
Annual relative humidity	0.007	[0.003, 0.013]
Elevation	0.013	[-0.013, 0.039]
Percentage forest coverage	2.482	[1.664, 3.317]
Percentage surface water coverage	0.036	[-0.215, 0.277]
Population density	-5.086 *×* 10^*-*5^	[-6.744 *×* 10^*-*5^, -3.367 *×* 10^*-*5^]
Median household income	-0.018	[-0.021, -0.016]
*Aedes aegypti*	-0.095	[-0.255, 0.059]
*Aedes albopictus*	-0.158	[-0.237, -0.071]
*Aedes canadensis*	0.185	[-0.039, 0.402]
*Aedes sierrensis*	-0.112	[-0.414, 0.204]
*Aedes trivittatus*	0.169	[-0.094, 0.414]
*Anopheles punctipennis*	-0.065	[-0.321, 0.182]
*Anopheles quadrimaculatus*	-0.076	[-0.246, 0.109]
*Culex quinquefasciatus*	0.099	[-0.099, 0.295]

**Table 3:** Parameter estimates from the reduced model

Parameter	Median	95 % HPD interval
Annual temperature	0.042	[0.027, 0.062]
Annual relative humidity	0.007	[0.002, 0.012]
Percentage forest coverage	2.599	[1.82, 3.473]
Population density	-5.177 *×* 10^*-*5^	[-7.074 *×* 10^*-*5^, -3.550 *×* 10^*-*5^]
Median household income	-0.018	[-0.021, -0.016]
*Aedes albopictus*	-0.165	[-0.246, -0.081]

Most of the significant factors have an intuitive interpretation. For example, the positive regression coefficient for the temperature and relative humidity factors implies that heartworm is more prevalent in warmer and humid locations. On the other hand, as is seen by negative regression coefficients, heartworm prevalence decreases with increasing population densities and median household incomes. Given the presence of relative humidity in the model, it is not overly surprising that precipitation drops out of the model fit. Finally, the negative regression coefficient on *A. albopictus* presence is not a contradiction: in the presence of all other factors (which include space and time prevalence histories), presence of this mosquito is associated with lessened heartworm prevalence. It is worthwhile to note that in a separate analysis (results not shown) a model with only *A. albopictus* was fitted, and the accompanying regression coefficient was non-negative.

**Fig. 3:**
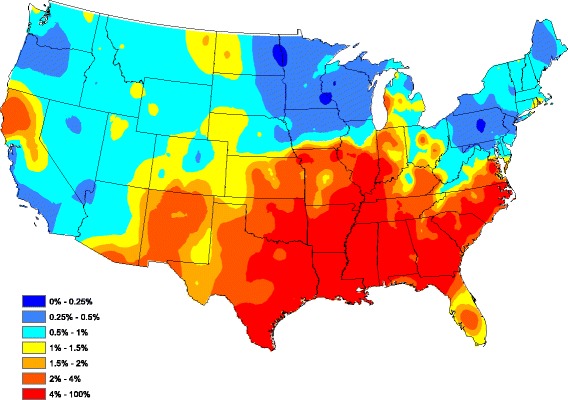
Model-based heartworm prevalence

To assess the overall performance of our reduced model, Fig. 3 graphically portrays our fitted model by plotting the average (over all 5 years) of model-estimated prevalence for each county after smoothing (standard Kriging with default parameters were used here). The model-estimated prevalence in Fig. 3 compares well to the head-banging smoothed baseline in Fig. 2. In fact, the correlation between the Figs 2 and 3 graphics is 0.727 (only counties reporting at least one test during the 5 year study period were used in this calculation). Clarifying further, our correlation between the two observation sets {*A*_*s*_}_*s* = 1_^*S*^ and {*B*_*s*_}_*s* = 1_^*S*^ is

6 $$ \mathrm{Corr}\left(\left\{{A}_s\right\},\left\{{B}_s\right\}\right)=\frac{{\displaystyle {\sum}_{s=1}^S}{n}_s\left({A}_s-\overline{A}\right)\left({B}_s-\overline{B}\right)}{\sqrt{{\displaystyle {\sum}_{s=1}^S}{n}_s{\left({A}_s-\overline{A}\right)}^2{\displaystyle {\sum}_{s=1}^S}{n}_s{\left({B}_s-\overline{B}\right)}^2}}, $$

where

$$ \overline{A}=\frac{{\displaystyle {\sum}_{s=1}^S}{n}_s{A}_s}{{\displaystyle {\sum}_{s=1}^S}{n}_s},\kern0.5em \overline{B}=\frac{{\displaystyle {\sum}_{s=1}^S}{n}_s{B}_s}{{\displaystyle {\sum}_{s=1}^S}{n}_s} $$

are the sample-size weighted averages of {*A*_*s*_}_*s* = 1_^*S*^ and {*B*_*s*_}_*s* = 1_^*S*^, and *n*_*s*_ is the number of tests conducted in county *s*. Since the correlation here is between smoothed and model-estimated prevalence (these are non sample size dependent quantities), the weights were taken as *n*_*s*_≡1 (and not the county-by-county sample sizes). The 0.727 correlation achieved indicates that the regression model has explained most of the data structure.

The fitted model has a number of uses. In the next section, it is used to construct annual heartworm prevalence forecasts. The model could also be used to estimate how climate change could alter heartworm disease risk.

### Forecasting

This section shows how to use our model to forecast next year’s regional heartworm prevalence. For this, all six significant explanatory factors and the spatial-temporal effects will need to be forecasted for the forthcoming year. To see how our forecast performs, the 2015 test and factor data was removed from the analysis, and the proposed six-factor model was refitted using data from 2011 to 2014 only. Our forecasts simply “plug in” 2015 forecasted factors for *A. albopictus*, annual temperature, annual relative humidity, percent forest coverage, population density, median household income, and a randomly generated random effect component into our model; for further details see [29, 30].

Two of the six factors are relatively stable over time: county forestation and the presence of *A. albopictus*. For these two factors, the most recent observations are used as 2015’s forecasted values.

To forecast annual temperature, historical temperature records were collected from 1895 to 2014 for each county and modeled as an autoregressive model of order one. The AR(1) model for an annual temperature series {*F*_*t*_} (previously denoted by {*X*_*s*,1_(*t*)} in Section 3 for county *s*) obeys the difference equation

$$ {F}_t=\delta +\gamma {F}_{t-1}+{\omega}_t, $$

where {*ω*_*t*_} is zero mean white noise; for further time series forecasting information, see [28]. The AR(1) model can be fitted to the temperature observations using practically any statistical software package. Let $\widehat{\delta}$ and $\widehat{\gamma}$ denote estimates of *δ* and *γ*, respectively. A prediction of the annual temperature at year *t* + 1 from temperatures from year 1 to year *t* is

$$ {\widehat{F}}_{t+1}=\widehat{\delta}+\widehat{\gamma}{F}_t. $$

**Fig. 4:**
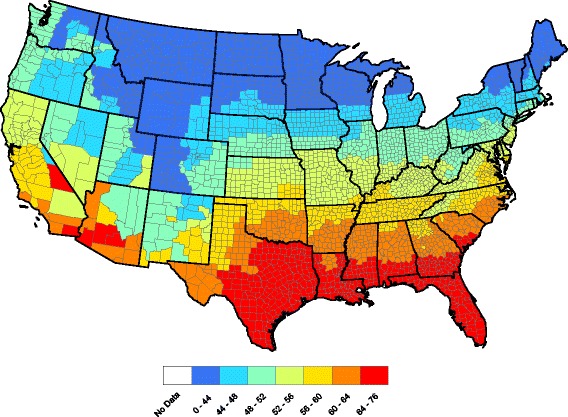
County-by-county forecasted 2015 annual average temperatures

**Fig. 5:**
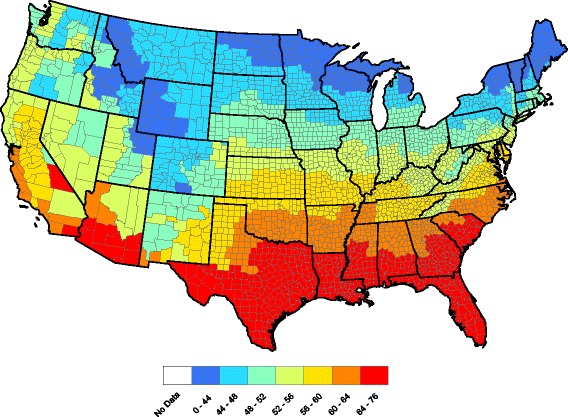
County-by-county observed 2015 annual average temperatures

In our forecast, ${\widehat{F}}_{t+1}$ is used as next year’s forecasted temperature factor. Figures 4 and 5 compare forecasted and observed annual temperatures for 2015. The correlation between these two figures in (6) is *r* = 0.996, which is very good (*n*_*s*_≡1 here).

A simple linear regression was used to forecast next year’s relative humidity (previously denoted by {*X*_*s*,3_(*t*)}) and median household income (previously denoted by {*X*_*s*,8_(*t*)}) in each county. Historical relative humidities from 2006 to 2014 and median household incomes from 1997 to 2014 were used to fit a regression model of form

$$ {I}_t=\alpha +\kappa t+{\eta}_t $$

for each county. Here, {*I*_*t*_} denotes the relative humidity (previously denoted by {*X*_*s*,3_(*t*)}) or median household income (previously denoted by {*X*_*s*,8_(*t*)}), {*η*_*t*_} is zero-mean random noise. Least squares estimators of *α* and *κ*, denoted by *α* and $\widehat{\kappa}$, respectively, were computed from the data at each county. The forecasted value for year *t* + 1 is simply

$$ {\widehat{I}}_{t+1}=\widehat{\alpha}+\widehat{\kappa}\left(t+1\right). $$

Forecasting the county population density for next year requires the county areas and their recent population counts. The US Census provides good county population estimates for 2010, but not in years since 2010. Estimated state populations were obtained for each state between 1969 and 2014. A simple linear regression was fitted to these data for each state and 2015 state populations were forecasted. This forecasted state population was then partitioned to the counties within the state at a proportion that agrees with 2010 Census proportions.

To forecast the next year’s spatial and temporal random effects, formula (3) is applied. Since the **ϕ**_*t*_ s are independent and identically distributed over various years, based on the values of *τ* ^2^ and *ρ* (available from the posterior samples), for the current time *t*, **ϕ**_*t* + 1_ can be generated from the multivariate normal distribution N(**0**, *τ* ^2^(**D** − *ρ* **W**)^− 1^). Then **ξ**_*t* + 1_ is calculated by **ξ**_*t* + 1_ = φ**ξ**_*t*_ + **ϕ**_*t* + 1_. This process is repeated for each value of (*ρ*, *τ* ^2^, φ), which are available from the posterior sample, thus yielding predictive posterior samples of the next year’s random effect **ξ**_*t* + 1_. See [29, 30] for additional detail on obtaining predictive posterior samples.

**Fig. 6:**
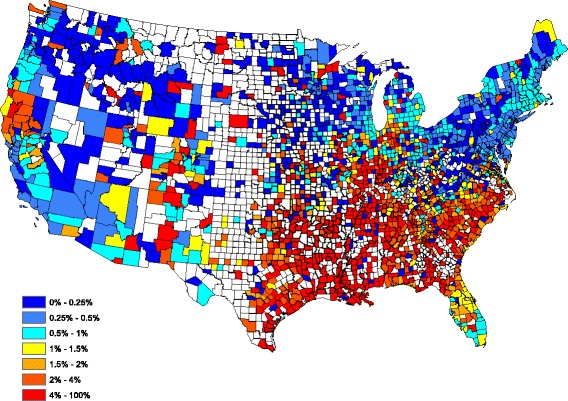
Observed heartworm prevalence for 2015

**Fig. 7:**
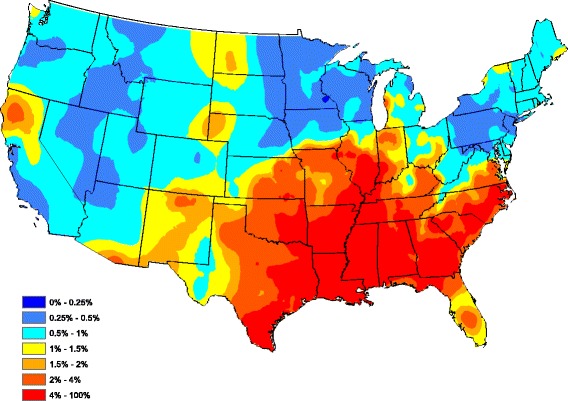
Forecasted heartworm prevalence for 2015

**Fig. 8:**
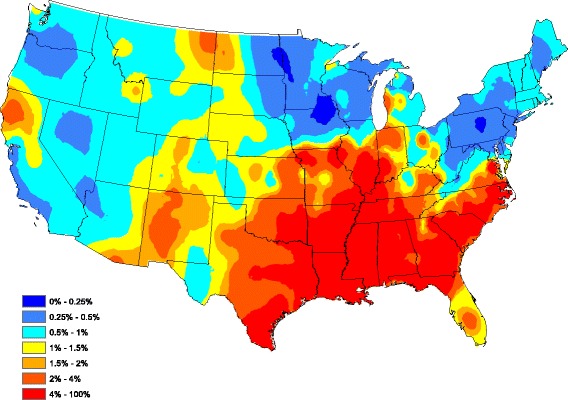
Forecasted heartworm prevalence for 2016

Figures 6 and 7 compare observed and forecasted heartworm prevalence during 2015. One can discern where heartworm is forecasted to be higher/lower than normal by comparing Figs. 2 and 7. The correlation between the Figs. 6 and 7 prevalence, as measured in (6), is 0.940. Hence, the model is accurately forecasting in locations that report more tests. Finally, Fig. 8 presents our heartworm forecast for 2016. When 2016 concludes, we will be able to compare this forecast to 2016 test results.

## Discussion

Generally, the management of emerging infectious diseases is approached reactively, with efforts focused on managing outbreaks after onset. The ability to reliably forecast transmission risk, particularly for diseases influenced by dynamic factors such as climate, could shift our paradigm from reaction to prevention. This is particularly true for vector-borne diseases, as specific environmental needs for vector survival are well documented [31]. One approach to infectious disease modeling is to use these factors to predict transmission and model the data in both space and time. This has been used successfully to estimate the incidence of malaria during eradication campaigns in Namibia and cutaneous leishmaniasis in high-risk areas of Columbia [32, 33].

Although preventable, heartworm disease is a relatively common and serious vector-borne disease of domestic dogs. Annual disease incidence, as reported by IDEXX and Antech, averages greater than 100,000 new cases annually. Annual data likely represent the true annual incidence of heartworm infection in domestic dogs: when diagnosed with heartworm, most dogs are either treated, or in some cases euthanized, due to poor outcome or financial constraints [34]. While fulminant infection with *D. immitis* may be due to lack of owner compliance in use of preventatives, it also may be due to misunderstanding the disease risk; mosquito vectors are known to be dynamic in their range and survival under changing climatic conditions.

To enhance veterinary client education and illuminate the benefits of preventatives, factors associated with *D. immitis* transmission [16, 35] were identified and used to develop a spatial-temporal conditional autoregressive forecast model of heartworm prevalence. A comparison of observed versus forecasted heartworm prevalence was made in 2015 and was quite accurate. This may be attributed, in part, to the fact that many of the factors influencing heartworm prevalence do not change significantly from year to year (e.g. forest coverage, population density, household income). While temperature and humidity change annually and are important disease risk factors [36], these factors are still reasonably predictable; however, environmental or climate catastrophes (e.g. regional climate shifts, flooding, hurricanes) could impact heartworm incidence. Finally, mosquito populations can fluctuate greatly from year to year as they depend on numerous local land-use and environmental factors. Some competent vectors of *D. immitis* are still expanding their range in the US [37].

Several of the mosquito presence/absence factors were not included in our final model; this may be because our currently available data are only presence/absence, whereas mosquito abundance, a purportedly more powerful factor, varies annually at a local level. More accurate mosquito counts would likely yield more accurate forecasts. In addition, human activities such as treatment abatement programs may impact mosquito abundances. Since the introduction of West Nile virus into the US, localities have developed or expanded mosquito control programs, including reducing breeding habitats and application of pesticides. With increased concern over the Chikungunya and Zika viruses, it is possible that increased mosquito control may be initiated in the coming year(s). If such programs are initiated, mosquito abundance counts should take these programs into account.

## Conclusion

In conclusion, our 2016 heartworm disease forecast (Fig. 8) has some noteworthy implications for veterinary practitioners, including an increased prevalence in northern California, eastern Montana, and central New Mexico. A relatively small increase in risk is predicted in some areas where heartworm is likely under appreciated, such as parts of the Dakotas and Nebraska. Importantly, our data indicate that all regions of the lower 48 United States have some risk for heartworm infection. Our maps and forecasts provide veterinarians with evidence-based recommendations for use of preventive in non-endemic regions of the US and support the recommendation of year-round use of preventive in high risk areas. Ultimately, we believe that these methods can be used to forecast multiple vector-borne diseases with veterinary and human health impacts, including Lyme disease, ehrlichiosis and anaplasmosis. Currently, the Companion Animal Parasite Council (CAPC, www.capcvet.org) provides monthly updates of heartworm prevalence on a county by county scale. Through a combination of real-time updates and forecasting efforts, we hope to see fewer cases of heartworm disease in dogs and cats in the future.

## Abbreviations

AR(1): First order temporal autoregression
CAPC: Companion Animal Parasite Council
CAR: Conditional autoregressive model
HPD: Highest posterior density
MCMC: Markov chain Monte Carlo
ML: Macrocyclic lactones
